# The Institutional Grammar Tool meets the Narrative Policy Framework: Narrating institutional statements in consultation

**DOI:** 10.1002/epa2.1126

**Published:** 2021-07-15

**Authors:** Claire A. Dunlop, Jonathan C. Kamkhaji, Claudio M. Radaelli, Gaia Taffoni

**Affiliations:** ^1^ University of Exeter Exeter UK; ^2^ Politecnico, Milan Milan Italy; ^3^ School of Transnational Governance EUI Florence Italy; ^4^ UCL London UK

**Keywords:** consultation, discourse, European Union, Institutional Grammar Tool, Narrative Policy Framework, narratives

## Abstract

We compare the Narrative Policy Framework (NPF) and the Institutional Grammar Tool (IGT). Given the focus of this special issue on the NPF, we first theorize how the IGT can contribute to the development of NPF categories, but also how the former gains conceptual leverage from the latter. We argue that it is useful to consider jointly NPF and IGT as this expands the benefit of NPF usage for policy researchers—uncovering not only the stories policy actors tell but also what these stories mean in terms of institutional statements. We provide a demonstration of how the conversation between these two policy lenses may develop by analyzing original data on the design of consultation procedures in the European Union, Finland, Ireland, and Malta.

## 摘要

我们比较了叙事政策框架（NPF）和制度语法工具（IGT）这两个理论视角在公共政策过程中的中心分析类型，这两个理论视角都与基于语言的分类紧密联系。鉴于本期特刊的重点是NPF，我们首先对“IGT如何能对NPF分类的发展作贡献”进行理论化，以及NPF如何能从概念上改进IGT。我们论证认为，将NPF和IGT进行共同衡量是有用的，因为此举能扩大NPF使用对政策研究者的益处—不仅能发现政策行动者的叙事，还能解释这些叙事在制度表述（institutional statements）方面的意义。通过分析有关欧盟、芬兰、爱尔兰和马耳他的咨询程序设计的原始数据，我们证明了IGT和NPF这两个政策视角之间的互动能如何发展。

## Resumen

Comparamos las categorías analíticas centrales de dos lentes teóricos sobre el proceso de políticas públicas ancladas en categorías basadas en el lenguaje: el Marco de Política Narrativa (NPF) y la Herramienta de Gramática Institucional (IGT). Dado el enfoque de este número especial en el NPF, primero teorizamos cómo el IGT puede contribuir al desarrollo de categorías NPF, pero también cómo el primero obtiene influencia conceptual del segundo. Argumentamos que es útil considerar conjuntamente NPF e IGT, ya que esto amplía el beneficio del uso de NPF para los investigadores de políticas, descubriendo no solo las historias que cuentan los actores de políticas, sino también lo que estas historias significan en términos de declaraciones institucionales. Ofrecemos una demostración de cómo se puede desarrollar la conversación entre estos dos lentes de políticas mediante el análisis de datos originales sobre el diseño de procedimientos de consulta en la Unión Europea, Finlandia, Irlanda y Malta.

## 1 INTRODUCTION

The Narrative Policy Framework (NPF) is one of the main theoretical lenses on the policy process. It approaches the study of public policy from the perspective of the stories that characterize policy controversies and, more generally, public policy (Jones et al., 2014; Shanahan et al., 2017). As an actor‐centered approach, the NPF theorizes that actors discursively portray all the major elements of public policy in narrative form. Policy narratives follow a common structure that can be identified empirically via different techniques of coding, experiments, and discourse analysis (e.g., see Gray & Jones, 2016; Jones & Song, 2014; O’Bryan et al., 2014; Shanahan et al., 2017).

In the context of this special issue, dedicated to the NPF (Stauffer & Künzler, 2021) our theoretical objective is to show if, and if so how, the Institutional Grammar Tool (IGT) can contribute to the development of the NPF. The IGT, we argue, provides the NPF with more precision and a granular understanding of how certain NPF categories, such as characters, plot, and moral of the story are set in institutional statements. It offers a new language to talk and explore narratives. We also observe that the IGT itself gains conceptual leverage from the NPF’s dynamic quality—especially in relation to the category of time. This means not only is the IGT a resource for NPF researchers, but also that a full conversation between the two approaches is feasible and productive. Policy researchers can be empowered by a navigation map that points to the bridges and transitions between NPF and IGT.

Moving from the theoretical objective to research questions, we explore the following two:

1. How do the NPF categories map onto IGT rule types? We theorize some possible plausible connections and probe our conjectures empirically;
2. Hence, our second question is empirical: considering an empirical unit of analysis, what do policy researchers gain from applying jointly the NPF and the IGT?

Our empirical analysis will concern consultation procedures about the formulation of new laws and regulations—also known as “stakeholder engagement tools” or “notice and comment.” These procedures are made up of rules—and, given their aim of communicating the government's intention to stimulate the engagement of stakeholders and citizens, they also have a narrative structure.

In the remainder, we first theorize how NPF categories travel into IGT categories (and back). Then, we develop our research questions. We address the questions with original data gathered on official nationwide guidance on stakeholder engagement in the preparation of primary and secondary legislation. Essentially, in our empirical section we demonstrate that a conversation can and should take place, and draw lessons in the Conclusions.

## 2 STARTING THE THEORETICAL CONVERSATION

Before we start, we need a few words (for the NPF‐oriented readers of this special issue) on the IGT, and, then a justification for the choice of comparing these two policy lenses. Like the NPF, the IGT moves from the analysis of policy language (Crawford & Ostrom, 1995; Ostrom, 2005). Developed within the broader Institutional Analysis and Development (IAD) framework by Elinor Ostrom and her associates (Ostrom, 2011; Schlager & Cox, 2017), the IGT captures the interactive dynamics of so‐called “action situations” by studying the grammatical features of institutional and governance arrangements. In these “action situations”, individuals have roles and take decisions in the context of the information available to them. Focussing on key decision points of action situations, IAD spotlights the ways in which rules—in‐form or in‐use—shape the alignment of individual and collective interests.

The starting point to investigate the action situation rests on the grammatical structure of institutional statements that govern interactions between actors. These statements are not the only attribute of the action situation, but also, for a policy analyst, they are the primary focus of attention. There are three types of institutional statements: rules, norms, and shared strategies. According to the ADICO (Attributes‐Deontics‐aIms‐Conditions‐Or else) categorization, they share a common core (Attributes‐Choice‐Conditions) but differ in the presence of Deontics (norms) and Or else (rules). Focussing on the latter, the IGT has also developed a semantic system of categorization known as rule types. Crucially, these statements, whether analyzed grammatically or semantically, represent shared discursive (Crawford and Ostrom used the adjective “linguistic”) entities that “describe opportunities and constraints that create expectations about other actors’ behavior” (Crawford & Ostrom, 1995; now in Ostrom, 2005:137). Behavior in action situations is theoretically predicted and empirically observed (Ostrom, 2005:33) by adding to the triad of rules, norms, and strategies the attributes of the biophysical world and those of the community.

The second question mentioned above concerns the case for considering the NPF and IGT categories (for broader policy theory comparisons, see Schlager & Blomquist, 1996). Why choosing these two lenses? Both are actor‐centered (narratives and statements are invariably uttered by agents). They also assume a structural dimension of public policy—by this, we mean that there are essential features that are not random and can be recognized across a variety of contexts, times, and places. In short, they share the belief that the regularities we observe in the patterns of human interactions revolving around policy problems can be explained by a universal signifier—or, to put it better, how actors within their contexts reproduce that universal signifier.

In the case of the NPF, the universal form (or structural property) is discourse. Actors shape policy via discourses that take narrative forms. At the roots of the NPF lies the *homo narrans*—narrative is the principal form of human communication which brings cognitive order to an otherwise chaotic social world. For the IGT, following Crawford and Ostrom (1995), the universal form is institutional language. Actors shape policy through the reproduction of rules‐in‐form and rules‐in‐use which set the boundaries of collective action or represent the rules of the interactive governance game. The universality of rules lies in being linguistic products characterized by universal grammatical (ADICO) and semantic (rule types) structures. The universality of the different articulations of language (expressed through narratives and rules which show common structural features across space and time) is therefore the common core which draws us to start this conversation.

Empirically, the unit of analysis of the NPF and the IGT varies (narratives and rules), but fundamentally they are drawn from language. The IGT creates observations and data via a grammatical/semantic approach, while the NPF considers that language is articulated in narrative structures. And the connection goes deeper: narrations do not fluctuate in vacuum. They are communicated by actors in situations governed by their institutional grammar. At the same time, institutional statements are not a given. Language is also the form in which people share meanings and make sense of institutional statements. Narration is a classic form in which individuals make meanings explicit and derive implications for their behavior.

We finally observe the conceptual and empirical agility of the NPF and the IGT. The NPF works with a structural template of how policy narratives appear in language. Political scientists have different options when studying policy narratives (Tuohy, 2019). The NPF is flexible enough to account for specific narratives that appear in a given policy controversy and broader narrations of an administrative process, a country's approach to a given problem/opportunity, an institution (Shanahan et al., 2017:180; 195–197) and narratives that “create socially constructed realities that manifest as institutions” (Shanahan et al., 2017:195). The IGT has an equally wide‐range of applications (Siddiki et al., 2020). Its flexibility for the analysis of a corpus of laws and regulations has been shown in previous research (Dunlop et al., 2019). A study has applied the IGT to the corpus of consultation in the 27 countries of the European Union and the UK (Dunlop et al., 2020).

Since this special issue is dedicated to policy narratives, we start our journey from four NPF concepts: setting, characters, plot, and moral. While there are many additional components and narrative strategies being added to the NPF as the field grows, these are central components in the literature (Jones et al., 2014:5–7). How can a policy researcher approach these categories with the IGT rule‐types?

The setting or context is the discursive representation of where the action is situated. For the IGT, the context is the core non‐biophysical attributes of the community organized by the institutions of the polity.

Next come the characters who are described using categories drawn from policy controversies. In controversies, actors take on specific roles that are discursively portrayed by dint of motives, normative qualities, and resources. Three classic NPF characters are heroes, villains, and victims (Shanahan et al., 2017; Stone, 1988). These are common because they nudge listeners and readers towards a given conclusion—for example, villains are motivated by wrong or ill purposes (Shanahan et al., 2017). Of course, they are not the only characters possible. Most notably, there is always a narrator (Schlaufer, 2018) —sometimes a named character (possibly the government or a central regulatory oversight body) but often a nameless but all‐knowing actor telling the story. Either way, we theorize that characters are found in the IGT by two rules: position and boundary. Position rules define the role of an individual or collective actor, while boundaries demarcate eligibility for the position. For example, an institutional statement may refer to “all citizens” or “those affected by this policy proposal” as actors who can contribute to public consultation.

Policy stories typically come with a causal plot. Plots can be coherent—with clear beginning, middle, and end, and attendant causal and temporal connections—but they may exist only in fragments where causation is non‐linear, segmented, even incoherent. When present in its most coherent form, the plot is the set of cause‐and‐effect mechanisms connecting past to present and future (Shanahan et al., 2017). We argue that in the IGT the plot will emerge as a combination of some (although perhaps not all) of the different rule types which are found at the heart of the institutional action situation. Choice rules define what actors can do in a given situation. Information rules refer to publication and transparency requirements. Aggregation rules have a place in the plot when two or more actors must convene and produce a collective decision, or when an actor is convened by another with authority to take a decision affecting the first actor. The plot may also include sanctions and rewards—“if you do this, you will find yourself better off and rewarded in the future.” For the IGT, these are payoff rules.

Finally, scope rules inform the audience of the aims of the narration and, within the broad narrative scheme, of the specific desired or prohibited outcomes of the action situation. We theorize that they should appear mostly in the NPF category of moral of the story. The moral assigns purpose to the actions of the characters. This is the “point” that the story makes. For an IGT scholar, the moral is a set of scope rules showing why it is good, efficient, and desirable to act in line with the institutional statements.

Before we close, we need to add the temporal dimension. While the NPF is explicit in considering time as a defining characteristic of the plot, rule types are static and do not openly include the time dimension—although scope rules implicitly refer to time as the aim to be achieved one day. Table 1 sums up these connections.

**TABLE 1:** Linking NPF categories to IGT rules

NPF	NPF definition	IGT	IGT definition
Setting Context of narrative development		Attributes of the community	Exogenous context
Characters Human or non‐human, individual or collective		Position	Identify positions/roles to be filled by actors (individuals or collective)
Characters Human or non‐human, individual or collective		Boundary	Regulate eligibility of actors to occupy positions
Plot Causal story linking past‐present‐future		Choice	Specify actions that actors must, must not, or may undertake
Plot Causal story linking past‐present‐future		Aggregation	Discipline actions or decisions that require the aggregation of two or more actors (e.g., rules about independent oversight)
Plot Causal story linking past‐present‐future		Information	Identify channels and modes of communication/exchange of information between actors
Plot Causal story linking past‐present‐future		Payoff	Assigns benefits and costs—for example, rewards and sanctions—to specific actors relative to following distinct courses of action
Plot Causal story linking past‐present‐future		Scope	Identify required, desired, or prohibited outcomes of the action situation. They implicitly refer to a time dimension
Moral The point of the story		Scope	Identify required, desired, or prohibited outcomes of the action situation

## 3 CASE SELECTION AND DATA GENERATION

Turning to case selection, we must identify an action situation where institutions speak about what actors should do and why. This leads us to the choice of institutional statements affecting policy processes, or rules contained in procedures. The institutional “speech” must also embed some form of narration rather than being a dry technical/legal listing of prescribed actions. An important characteristic we look for (in order to endogenize time) is the sequential nature of the action situation—something typical of regulatory and administrative procedures.

Consultation has a prominent procedural aspect (hence it has potential for IGT analysis) but underpins also a specific ideological approach to policy making which very often warrants guidelines rich in examples, stories, and causal plots. Consultation is new territory for the NPF because it does not belong to the field of policy controversies. Governments publish consultation guidance not to engage for or against an option, hence we may not expect heroes, villains, or victims in the standard sense. However, they can still exist in slightly different forms: the government can be the knowledgeable narrator with heroic qualities; stakeholders can be described in positive terms, as individual or collective entities that can provide evidence and broaden the views and legitimacy of the policy‐makers; bureaucracy, and red tape can appear as villain. The IGT, and more generally the IAD, have often considered common pool resources, therefore, consultation is new territory for the IGT too.

Empirically, IGT rules were gathered using protocols that identify the exact wording of a rule in primary or secondary legislation on consultation in force at the time of data collection (2018–2019). These original data on law as text were gathered for all European Union (EU) countries and for the guidelines on consultation of the European Commission (2017)—an organization with its own policy formulation process. Lawyers based in each country were hired on a temporary basis to assist with the correct identification of the legal base in force in 2018 and the retrieval of IGT rules—in original language and English translation.

Having computed the total number of consultation‐related rules for each case, we selected cases on the basis of density of rules (hence ignoring the fourth quartile with a low number of rules), geographical spread, and practical constraints (resources). Our cases are from the first, second, and third quartile. Our reasoning is that cases with a small number of rules (Austria, Belgium, Czech Republic, Denmark, France, and the Netherlands) indicate the lack of interesting narrative features (the story is very short) or lack of formal consultation—few institutional statements in formal guidance point to either symbolic or informal procedures of stakeholder engagement.

We selected the cases of the EU and Ireland from the first quartile, Finland from the second quartile, Malta from the third. The presence of the EU is interesting also for the multi‐level character of this organization. However, consultation and more generally the better regulation policy and tools of the EU, although somewhat influential, do not prescribe the design of consultation in the member states. The OECD data (2018) show that variability is still considerable—in the OECD data, the closest country to the EU in terms of better regulation procedures is often the UK, which has now left the EU. Furthermore, while several member states have an administrative procedure act, there is no legal text defining an EU administrative act—hence it is impossible to theorize a top‐down influence of the EU on administrative procedures. Diffusion has originated primarily from the OECD, not the EU (De Francesco, 2013).

The data on narrative categories were gathered by coding the consultation guidance with an NPF template. Again, this is because the special issue is dedicated to the NPF, and therefore this lens ought to be our point of departure. We coded relevant portions of text included in guidance documents using the NPF categories of Table 1. Furthermore, for each portion of text belonging to an NPF category, we identified the correspondent IGT rule type(s). Two authors coded independently the same case to check on construct validity and reliability (pilot stage). Then after having finalized constructs, each author generated the final data on a single case.

We now take each case in turn—following the setting‐characters‐plots‐morals NPF format. Each case also contains a summary table for easy reference to the connections between NPF and IGT categories.

## 4 EMPIRICAL ANALYSIS

### 4.1 European Union

#### 4.1.1 Setting

The EU Guidelines on Consultation (hereafter the Guidelines) are set in the context of a monumental Better Regulation Guidelines (they make up the seventh chapter, European Commission, 2017). Consultation is narrated with a grand‐angle, not only as part of the Better Regulation policy, but also as an overarching activity that informs all the stages of the EU policy process, from inception to evaluation. In setting the stage, the Commission distinguishes between formal consultation and more generic feedback: “Consultation involves a more structured engagement with stakeholders where the consultation principles and standards apply; whereas the feedback process allows stakeholders to provide comments on a particular document which will be considered in the further elaboration of the document or initiative” (European Commission, 2017:70, footnote 105).

Formal consultation applies to impact assessments but also to evaluations, communications, and green papers. Feedback is envisaged for other activities such as providing views on draft legislation. Boundaries define the scope of application of the procedures. Since in IGT terms, boundaries are about the eligibility of an actor to take on a position, the exclusion clauses belong to the setting rather than characters. Talking about exclusion clauses, consultation does not interact with the participatory process of the European Citizens Initiative and the neo‐corporatist process of consultation of social partners.

#### 4.1.2 Characters: the narrator

The first character is the narrator. Who tells the story? It is the Commission. This character gives the cards to the players and defines authoritatively the subject matter: “stakeholder consultation is a formal process by which the Commission collects information and views” (European Commission, 2017:68). We find a voice that is formal, prescriptive, and of legal intonation. The narrator puts emphasis on the formal nature of consultation and distinguishes it from the more generic provision of feedback. The register is definitively top‐down and prescriptive—“should” appears 61 times in the document. Although the Guidelines open with a sentence referring to the simplicity of consultation (European Commission, 2017:67), the reader is warned that consultation is mandated by the Treaty (art.11) and Protocol no.2 (on subsidiarity and proportionality) annexed to the Treaty. As with most of the activities of the Commission, consultation is narrated via the language of legal requirements and steps that are mandated, prescribed, and must take place. Well, after all this is the world of *formal* consultation—the reader has been warned right from the start.

And yet, there is a second register in the voice of the narrator. This is the register of tools, techniques, methods, and smart ways of operating. It is in a sense reminiscent of the new public management register—the language of tools that make organizations smarter and capable of learning, as well as open to the world of affected interests. Previous work has indeed connected the late wave of new public management to the better regulation agenda (Radaelli & Meuwese, 2009). Finally, in one case, the narrator is unnecessarily humble—in contradiction with the other registers. This happens when we read in the introduction that the officers “should read these guidelines” (European Commission, 2017:67). Certainly, the Commission expects the officers to implement the Guidelines, not to simply read them.

#### 4.1.3 Characters: the lead service and the stakeholders

The second character we find is the officer, defined by position rules. Those who carry out consultation are asked to conduct an impressive range of activities, keeping the whole exercise, balanced, open to different voices (from experts to religious communities), information‐rich, useful, and accountable. If this is not a hero, it is definitively someone with extraordinary commitment to the cause of level‐playing field in consultation.

The Guidelines are directed to “officials” and “managers” but seems to prefer the language of “lead service” in charge of developing the consultation. This collective dimension is important also because at least in the case of consultation within impact assessment processes, the Commission rolls out the various activities via an inter‐service group which includes the lead and the most concerned Directorates General and the Secretariat General. It is indeed the Secretariat General that is responsible for launching all public Internet‐based consultations.

The third character is the stakeholder. This has some of the properties of the hero—it is the stakeholder who provides views, input, and informative evidence. However, the Guidelines also warns these heroes can turn into villains when they capture the Commission's officers (European Commission, 2017:76), orchestrate consultation campaigns (p. 78), and when they pursue “special interests” (p. 68) as opposed to the “general public interest” (p. 68). Incidentally, the Member States are also sort of villains when they purse their “particularistic interests” (European Commission, 2017:68).

#### 4.1.4 Plot

The plot starts from the acknowledgment that formal consultation is a duty. Taking this duty seriously leads officers to inform policy with evidence, and improve on the legitimacy of EU legislation. If we now read the plot in IGT terms, we find that it is made up of a combination of rules (mostly choice, information, and scope rules). The scope rules define the central trajectory. They are defined as four general principles of: participation, openness and accountability, effectiveness, and coherence. These central aims are achieved in the context of the minimum standards of clarity, targeting, publication, time limits, and acknowledgement of feedback.

Let us now consider the rules that kick‐off the plot in detail, starting with three broad choice rules: establishing the consultation strategy; conducting consultation work; and informing policymaking. In turn, each of the three phases contains additional rules: four on establishing the strategy (three choice rules and one information rule on the creation of the consultation page), four on carrying out the work (a mix of choice and consultation) and two choice rules on informing policymaking (synopsis report to support decisions and provision of feedback, which can also be considered an information rule). The narrator holds the hand of the officer, painstakingly explaining in detail what the stages of consultation are and the specific rules of each stage. To illustrate: one rule contained in the rule “establishing the strategy” is to “set the objective of consultation”. But the rule about the objective is then further de‐composed into a mix of five choice and information rules. And each of these five rules opens up a new set, for example, the rule about establishing the context and scope of consultation includes five additional choice rules.

In this extremely dense rule‐bound environment, the Commission concedes that all rules must be adapted to circumstances (not ignored or bypassed, but customized). Thus, on the one hand, all the rules are described clearly and in detail. On the other, the narrator warns the reader that there has to be a degree of customization.

As for aggregation rules, the consultation strategy cannot be set independently by the lead service. It must be endorsed by the interservice group established for the policy initiative. The Commission draws on the beliefs and perspectives of different Directorates General to shed different lights on the monitoring process. This is in line with an approach that encourages a pluralistic process in policy formulation within the Commission, to break down silo mentalities (Radaelli & Meuwese, 2010). However, there is also an element of threat in the plot: officers should invest time in consultation otherwise there may be problems later—legal or otherwise: “[E]arly consultation can avoid problems later and promote greater acceptance of the policy initiative” (European Commission, 2017:68).

The rules interact and become a plot through the category of time. Time is essential to provide narrative dynamism to actions and their consequences. Thus, the IGT is a fine toothcomb when it explains the plot as a constellation of rules. But, the NPF reminds us that rules are played within a temporal narrative evolution. Time is about the steps in the process. Officers are told what comes before and what comes after. The whole consultation guidance has a sequential nature.

#### 4.1.5 Moral

What is the final purpose of consultation? What is the reasoning behind it? The starting point of the moral is about benefits:

The initial design, evaluation and revision of policy initiatives *benefits* from considering the input and views provided by citizens and stakeholders, including those who will be directly affected by the policy but also those who are involved in ensuring its correct application. Stakeholder consultation can also improve the evidence base underpinning a given policy initiative (European Commission, 2017: 68, our emphasis).

Thus, there are benefits not only for stakeholders but also for bureaucracies involved in policy delivery. Legitimacy (of EU legislation) is not featured explicitly. Instead, we find the word “acceptance” that evokes an authority‐subject relationship. Table 2 summarizes our findings.

**TABLE 2:** Consultation guidelines of the European Commission: NPF and IGT compared

NPF	IGT	Findings
Setting	Attributes of the Community—Polity	Consultation as activity carried out across the whole life cycle of EU policy
Characters	Narrator	Two registers: prescriptive/legal and managerial
	Other characters	Lead service Stakeholders Secretariat General
	Boundaries	Boundaries applied to the process, not to characters
Plots	Choice, Information and Aggregation	Thick web of nested rules across the sequence of consultation activities
	Payoffs	Not explicit sanctions, the threat is implicit: without meaningful consultation, there will be problems later on
	Scope	General principles Minimum standards
Moral	Scope	Benefits arising out of evidence‐based policy. Benefits fall on stakeholders as well as those concerned with policy delivery Benefits in terms of acceptance of EU policy

### 4.2 Finland

#### 4.2.1 Setting

Consultation is set in the context of Finland as a multi‐language state. That Swedish, Sámi‐speaking, and sign‐language minorities must be included along with the Finnish majority is a reference point of the document which features in *every* section of the guidance. Consultation issues are also set against Finland's broader international obligations—with the extension of the guide to cover the preparation of national laws for the implementation of EU legislation and international agreements (Government of Finland, 2016: section 1.1). Yet, when we compare this single mention to the continual reminders of the diverse linguistic terrain policy officers must traverse, we can say that the consultation setting for Finland is sovereign and local as opposed to international, possibly because Finland is a standard‐setter in terms of citizens engagement and participation.

#### 4.2.2 Characters

Finland's consultation guidelines grant positions to all the expected characters: government (and its departments and agencies); public sector bodies; the civil service; organizations; citizens; stakeholders; experts, and companies (Government of Finland, 2016). These usual suspects are passive characters, however; name‐checked as potential participants and affected parties but not given any narrative distinction. Rather, there are two sets of characters worthy of that description: minority citizens and desk officers. We discuss them below.

Minorities are potential victims that deserve special protection in the consultation guidelines. Indeed, over 10% of the document (in terms of words) is given over to linguistic inclusion of some form (see especially Section 4). Beyond the protection of linguistic minorities, the experience of different age groups, gender, ethnic minorities and the disabled are all noted (Government of Finland, 2016, for example, sections 2.2.3; 3.2.1 and 3.2.2).

These minorities deserve “special attention” given that they are “at risk of being excluded” (Government of Finland, 2016, section 2.2.3). But, how can we be sure this is not some cosmetic exercise? Two features of the characterization offer assurance to minorities as potential victims. First, the individual needs of each minority group regarding consultation are worked out and, on occasion, a minority group is broken down further into smaller sub‐groups (in particular regarding language). Thus, the common pitfall of assuming homogeneity of minorities is avoided. Second, the boundaries of these categories are delineated with great care (Government of Finland, 2016: sections 4 and 5). Such care suggests a sincerity of mission that goes beyond symbolic name checking.

Now to the second major protagonist in this story. The desk officer or the “drafter”, as they are referred in the document, is assumed to be the primary reader. This assumption is not simply a matter of logic—although we are on safe ground since these guidelines do serve as a “how to” for those bureaucrats designing consultations. The narrative is punctuated by moments when the drafter is spoken to almost directly. Most frequently, they are offered advice and reminded how important their actions are for the success (or otherwise) of the work: “[T]he presence and commitment of the drafter is important: it is especially needed in discussion and summaries” (Government of Finland, 2016: section 3.7.2). More prosaically, the drafter is reminded that they personally should be associated with the consultation with their contact details on the website.

The document's author is never revealed. *Säädösvalmistelun kuulemisopas* (Guide to Legislative Consultation) is hosted by the portal of the Finnish legislative database, Finlex. Fi hence we know the narrator is the government. However, the text does not make references to this actor, for example, we did not find sentences like “the government instructs officers to….” The narrator is a shadow character: always there but unidentified (although officers realize it is the government who is speaking). This narrator is omniscient when it comes to consultation. And, much like in ancient allegorical tales, the narrator's purpose is to supply the clear pedagogical voice that runs throughout the story. This voice takes two forms: the pedagogical preacher and the pedagogical teacher.

The preacher aims to sell the vision of what consultation in its best form (i.e., inclusive) can achieve for the content of the policy and its wider social legitimacy. For example,

[T]he principle of transparency must always be borne in mind when consulting and planning it. When using different methods of consultation, equal treatment of stakeholders and citizens must always be ensured … Consultation and communication must always have a goal. Often the objectives are primarily related to information needs, but the importance of interaction in the process as such should not be forgotten. Interaction increases trust (Government of Finland, 2016: section 2.2.2, emphasis added).

The narrator also plays the traditional pedagogical part of teacher, instructing the desk officer on the nuts and bolts of “how to” run an inclusive consultation—which are supported by copious exemplars in annexes. The teacher voice is unmistakable. The instructions lack equivocation, certain tasks are frequently marked as “important” and “essential” and the consequences of taking short cuts are spelled out.

#### 4.2.3 Plots

Constructed using essentially choice and information rules, Finland's consultation guidance plot structure contains one central master plot which is supported and elaborated with three sub‐plots. Taking the master plot first—expressed through choice rules—the core causal story imparts the rational vision of evidence‐based policy‐making (EBPM). By undertaking a certain set of clear analytical steps—which mirror the traditional idea of the policy cycle (Government of Finland, 2016: sections 1 and 2) —consultations lead to a scenario of better policy results and social legitimacy (scope rule).

This EBPM master plot is closely supported by a more detailed sub‐plot on precise “how to” instructions. At points, the guidelines read like a “101” methods guide. The document is full of exemplars and ideas about running a consultation and pitfalls with methods. Importantly for the NPF, these information rules are always linked back to a teacherly explanation of why they matter for the success of the consultation, for example, this is the path to inclusivity and this is how you (officer) deal with the volume and diversity of stakeholder inputs that you want to encourage.

Four information rules are tied to the positive scenario that is the end‐point of the plot: clarity, minority languages, communication medium, and meaningful consultation methods. These also hint to a (weak) payoff rule where “[P]roceedings may be delayed if the documents are not available in both Finnish and Swedish” (Government of Finland, 2016: section 4.2). There are no direct sanctions, but officers are penalized by seeing the proposal stuck in delay.

This lesson in the art of convening is supported by a further sub‐plot on the temporal dimension. Structured with choice and information rules, time is presented as central to the success of inclusive, fair, and evidence‐based consultations. Specifically, time is conceived of in a sophisticated way—it is multi‐dimensional and should be understood from the stakeholder's point of view rather than tied to the timetable of the desk officer. The guidelines discuss six dimensions of time that are mission critical—upstream; during; untimetabled time; exceptional circumstances (e.g., holidays); planning time throughout the consultation life course and finally feedback (Government of Finland, 2016: sections 1.4, 3.3, 3.6, 1.7–1.9, 2.3.2, 3.2.2). On feedback, the need to close the feedback loop with stakeholders is continually referenced.

There is one final sub‐plot that supports the EBPM causal story: warnings that shortcuts should be avoided. We find many moments where the temptation to deploy a shortcut is anticipated and warned against, such as: “multi‐member preparatory bodies are not a substitute for other consultations, which provide an opportunity for non‐preparatory parties to participate and influence what is being prepared” (Government of Finland, 2016: section 3.2.1). These warnings address two themes about preventing cosmetic exercises: (a) inclusion being attempted in a comprehensive way and (b) consultations’ timelines being open enough to allow real participation. Note, the warnings do not tell us about any sanctions or reward.

Wrapping up, there is a learning model implicit in this plot—our teacher narrator gives the desk officer clear instructions but these are always accompanied by an explanation about the logic behind those instructions, their multiple temporal dimensions and the obvious shortcuts that will undermine the consultation.

#### 4.2.4 Moral

Throughout the guidelines, the possibility that inclusive consultations (if they follow the rational EBPM master plot) can create the conditions for fairness and trust are continuously evoked:

The aim of the consultation is transparency and good quality in the preparation of legislation. The purpose of the consultation is to identify the various aspects, implications and practicalities of the matter to be prepared. Consultation enhances confidence in democratic decision‐making and legislation and promotes compliance with standards. The consultation will also strengthen the realization of civil and political rights (Government of Finland, 2016: section 1.2).

The acceptability of decisions improves with the experience of inclusion, and being consulted engages stakeholders not only in preparation but also in implementation and monitoring (Government of Finland, 2016: section 3.1)

Here, we have the preacher voice of our narrator selling the vision of what consultation in its best form (i.e., inclusive) can achieve for both the content of policy and social legitimacy. This is a kind of promissory narrative (see the sociology of expectations literature, especially Brown & Michael, 2003): fairness (equality and transparency) in the consultation has emancipatory potential for policy and participants. Table 3 summarizes the findings.

**TABLE 3:** Finnish consultation guidelines: NPF and IGT compared

NPF core features	IGT rules	Findings
Setting	Attributes of the Community—Polity	Inclusion of minorities (especially linguistic) International obligations
Characters	Positions Boundaries	Usual suspects—government and civil society policy actors Potential victims—minorities Reader—drafters Narrator—the government. But the government as such is not named explicitly. The narrator is nameless, omnipresent, pedagogical
Plots	Choice Information	Master plot—evidence‐based policy approach (EBPM) to consultation yields inclusive and fair results Sub‐plots—“how to” guides; time is multi‐dimensional; shortcuts can lead to failure
	Scope	Good communication practices generate and end‐point of the plot with better policy and social legitimacy
	Payoffs	Failure to respect language rights may delay procedures
Moral	Scope	Inclusive consultations create social legitimacy and trust

### 4.3 Ireland

#### 4.3.1 Setting

Ireland”s consultation guidelines are part of a major effort to modernize governance. The document, significantly entitled “Principles and Guidelines”, is conceived in the setting of wider governance reforms about establishing a “legislative footprint” to track legislative initiatives, consultation, publications of draft bills, pre‐legislative scrutiny by Parliamentary Committees, submissions received, and meetings held with stakeholders. Another reference is to the “Principles and Guidelines” as implementation of the review of national and international practice to develop engagement and consultation with citizens, civil society, and others by public bodies (DPER, 2016:3). The settings are well demarcated by the metaphor of the legislative footprint and the reference to international good practice. Like in other cases, boundaries do not refer to the eligibility of the actors, but to exclusion clauses—when consultation is not applied.

#### 4.3.2 The main character: the narrator

The narrator is the main player with a position above all the other characters. It is the narrator that defines the rules of the game. We find a narrator that instructs in a prescriptive and top‐down manner, the word “should” appears 65 times. The reference to the Principles (note the scale of ambition: this is not just Guidance) provides a sort of *gravitas*. Despite the solemnity of the narration, the narrator does not speak with a legalistic tone. Adjectives such as “clear, real, meaningful, proportionate and genuine” reveal a narrator that is prescriptive but not formal. Indeed, on three different occasions, the narrator starts a sentence with the term “Ideally” and “It may be best”—expressions that suggest an attempt at informality. The voice of the narrator emphasizes also other aspects that do not have a legal intonation, such as the “footprint” metaphor.

#### 4.3.3 Other characters

The second character is impersonated by those officers who have to be educated. The government and the departments are considered as a single main character and the guidance sets obligations that the characters must follow. “Governments should”, “The Department will”, “Officials should”.

The third character is represented by the stakeholders which are however somewhat a peripheral presence. The narrator never refers directly to stakeholders. They are part of the narration, but their role is limited to those who are assisted—“involving stakeholders” (DPER, 2016:12) is the classic expression related to this character.

#### 4.3.4 Plot

Consultation delivers a plot of systematic engagement and efficiency—because consultation can “reduce the burden of engaging with Government on policy development and implementation” (DPER, 2016:6). The plot has a higher‐level plane concerned with “a greater sense of political efficacy”, “confidence” (DPER, 2016:4) and legitimacy to achieve real world impact and knowledge sharing—this is the language of a solemn plot.

Scope rules are, in fact, plentiful. Consultation should be genuine, meaningful, timely, balanced, and with the ultimate objective of leading to better outcomes and greater understanding (by all affected interests) of the benefits and consequences of proceeding with a given policy proposal. The plot leads to a scenario of “real, meaningful, and targeted engagement” (DPER, 2016:3).

Apart from scope rules, the plot revolves around choice and information rules. Amongst these rules are those about the identification of the stakeholders, the decision to proceed, to receive, and to analyze feedback and review the consultation process. The rules extend to the broader activities that link consultation to the legislative footprint, lobbying, and the treatment of personal data. Information rules cover the publication of submissions and the provision of feedback.

The plot can be summarized as follows: consultation is a systematic process of meaningful engagement with those outside the policy‐making process that support the evidence‐base of the process. The final scenario of the plot is one of political efficacy, but there are also fundamental good governance outcomes such as confidence and trust in legislation. Citizens benefit from the wider, open knowledge‐base of policies, and awareness of how decisions emerge. The plot is reinforced by the dangers of non‐correct procedures, a payoff rule: “Officials should be mindful of the need to consult with each other to avoid creating cumulative or overlapping regulatory burdens” (DPER, 2016:6).

#### 4.3.5 Moral

The moral of the story is that consultation, beyond its benefits in terms of EBPM, has a point in terms of diffusing “a culture of innovation and openness by involving greater external participation and consultation” (DPER, 2016:4). Table 4 presents our findings.

**TABLE 4:** Ireland's consultation principles and guidelines: NPF and IGT compared

NPF	IGT	Findings
Setting	Attributes of the Community—Polity	Consultation is part of the movement towards good governance, the introduction of a legislative footprint and convergence with international good practice
Characters	Positions	Narrator is prescriptive but not legalistic Government, Department, Officials, Public Bodies Stakeholders (not addressed explicitly)
	Boundaries	Applied to the process (exclusion cases)
Plots	Choice Information	High number of choice and information rules describe the process leading to meaningful consultation
	Payoff	Lack of inter‐departmental consultation leads to the threat of regulatory burdens (the sanction appears ex‐post, with the emergence of red tape that damages the acceptability and efficiency of regulations)
	Scope	Real, meaningful, targeted engagement
Moral	Scope	Meaningful consultation promotes a culture of innovation and openness

### 4.4 Malta

#### 4.4.1 Setting

The setting of consultation in Malta is not particularly wide as the procedure does not apply, as an obligation, to all legislative or regulatory initiatives and proposals. Consultation, indeed, is employed on a case‐by‐case basis drawing on ministerial discretion. To use the guidelines’ wording, ministries are not bound by consultation (OPM, 2011: p. 8) and so enjoy a great deal of discretion on whether launching it or not. Once a government entity decides to launch a consultation exercise, although, the guidelines are very specific.

#### 4.4.2 Characters: one dominant position

As a result of this specificity, the intonation of the narrating character is formal, prescriptive, and top‐down, but it is not engrained in legal requirements (as in the case of the European Commission) —rather in procedural advice. In contrast to Finland, the narrator, while elaborating on the typologies of consultation, does not address and engage directly all the other characters involved in the exercise (stakeholders, minorities, and marginalized groups) but, like in Ireland, talks only to the main character of the story, that is, the considerate civil servant.

Although they elaborate on other characters while unravelling the main plot, the narrator puts herself in a dialogic relationship only with the government entities she aims to instruct by narrating the consultation tales. This is corroborated by the very fact that consultation guidelines are not publicly available through a governmental website. This makes the whole document and its narrative somewhat esoteric. It is a clear indicator of the internal use of the guidelines—and of the narration therein. Finally, stakeholders and societal actors do not hold specific rights to be heard. In IGT terms, the stakeholder position is created but choice rules are always those of the public administration.

The focus on government entities is reflected by the paucity of clear boundary rules. Boundary rules are absent because the decision of what stakeholders to engage is a discretional one of those who carry out consultation. As mentioned, consultation is not mandatory, and thus setting boundaries around a non‐requirement is unnecessary.

#### 4.4.3 From parameters to plot

It is telling that the Maltese document is called “Parameters”—in stark contrast to the solemn Irish “Principles”. A parameter defines an activity or the conditions of operations of a system. A rule prescribes behavior. Since the narrative is centered on one dominant character, the choice and information rules are the parameters to be considered by the considerate civil servant. The inward‐looking nature of consultation shows also in the fact that the few aggregation rules that exist cluster together governmental actors (inter‐institutional consultation) rather than stakeholders, citizens, and minorities.

Three sub‐plots, which belong to the main “consultation exercise” plot, are carefully narrated. These subplots are national consultation, sectorial consultation, and restricted consultation. Once selected a path, the narrator prescribes its steps for the government entities, but always in the context of a discretionary procedure.

This leads us to the logic of consultation guidelines in Malta, that is, educating government entities about the paths (and plots) to successful consultation while using a sub‐plot of embarrassment as a warning:

Before commencing an external consultation exercise, it is important that the issue being discussed is researched in order to be in possession of the best information, It can be very embarrassing for a Ministry or Entity and ultimately all of the Government to present an inaccurate or outdated policy, which will be highlighted during the consultation process (OPM, 2011: Section 04)

The presence of this sub‐plot, where the uninformed governmental entity is implicitly and emotively portrayed as the possible villain, allows us to advance two considerations. First, the potential hero of the consultation tale is the careful civil servant who conducts sound consultation exercise as per the guideline and hence is not unprepared and embarrassed vis‐à‐vis the stakeholders. This is reflected in a plot whereby the main agent is always the public entity which is addressed in a genuine “how to” style by the guide (tables, flowcharts, tips, and examples are all deployed). Second, the narrator impersonates the role of the preacher (as we found in Finland) who puts forth cautionary tales and warns the main character about the mistakes to avoid and the best practices at hand. This is exemplified also by the presence, disseminated throughout the document, of a series of boxes, cases, and examples that sound and work like parables or edifying examples, representing a parallel sub‐plot along the three main sub‐plots.

Importantly, the main character—our hero—is clearly the government entities which will carry out consultation in practice, whereas for instance in the Finnish case space is also made for minorities and stakeholders, with the public administration (PA) working on their behalf. In Malta instead, the guide serves the purpose of instructing the PA while the benefits to the stakeholders seem to be ancillary or a by‐product of the action of well‐conducted civil servants.

#### 4.4.4 Moral

The moral is that by following the informed guidance the considerate public manager will conduct successful consultation. Success is measured mainly by the adherence to the requirements of the guide itself rather than by the satisfaction of the stakeholders. The latter are broadly epiphenomenal to the narrative which sees the narrator and the hero in a strict dialogic relationship—other agents covering only ancillary roles. This is also reflected by the fact that the main beneficiary of consultation is the PA itself which, by following the wise advice of the narrator, is capable of extracting the most evidence‐rich stakeholders’ feedback and hence help the government achieve the best policy making solution. Or, to put it differently, good consultations ensure more transparency which increases the acceptance of government initiatives. Benefits are always inward‐looking. The main findings are summarized in Table 5.

**TABLE 5:** Malta's parameters for consultation exercises with stakeholders: NPF and IGT compared

NPF core features	IGT rules	Findings
Setting	Attributes of the Community—Polity	The guide, called *Parameters*, has an internal use, hence the setting is limited to an intragovernmental space although the role of consultation should be to open government up
Characters	Position	The narrator does not engage directly with the characters
	Boundary	Narration is inward‐looking with the diligent officer as hero The Parameters do not set boundary rules
Plot	Choice and Information	Plot is deployed in a context where consultation is not mandatory. Hence it focuses on the attractiveness of carrying out dutiful, diligent consultation Three sub‐plots: national, sectoral, and restricted consultation
Moral	Scope	The prescribed outcomes benefit in the first place the main character with the expected benefits of consultation for the stakeholders coming as by‐products

## 5 CONCLUSIONS

Our two research questions were about the mapping of NPF categories onto IGT rule types and benefits for policy researchers to consider jointly the two frameworks. The mapping exercise worked well: we theorized the correspondence between NPF categories and IGT rule‐types, and then carried out the journey from one lens to the others empirically.

Our exercise shows that the dialogue between the NPF and the IGT is possible. The NPF researchers benefit from the granular, systematic, rule‐oriented approach of the IGT. Thanks to the IGT potency, NPF actors can be empirically studied for what they *do* (beyond their normative attributions of heroes or villains or narrators): they produce and exchange information, perform key actions, distribute sanctions and threats, convene in decision making moments. The plot can be decomposed in different rule‐types, that click together to generate narrative steps, and lead the characters to a destination point in the story.

At the same time, the IGT researcher can benefit from the conceptual and empirical leverage of the story. Institutional statements define rule‐types that are then narrated—and via narration the action situation takes on its moral and normative quality, assigning blame to some and hero‐like attributes to others. With the NPF, we also find out more about the identity of the narrator (the sympathetic teacher, the instructor who knows the tools, the legalistic guide) that lies down the institutional statements. This IGT position (the narrator) takes on the colors of a particular identity. The narrations of consultation in the four cases are different across the cases. Incidentally, this also shows that the EU influence on the design of consultation in the member states is still limited. Table 6 illustrates these findings.

**TABLE 6:** Narrative elements across the four cases

	European Union	Finland	Ireland	Malta
Setting	A web of nested rules	A pedagogical narration	A governance narrative of principles, values and tools	A self‐referential narrative of parameters
Narrator character	Prescriptive‐legal but also with new public management vocabulary	All‐knowing preacher and teacher	With gravitas, the narrator lays down the map of principles and tools	Formal, prescriptive, top‐down but in a context where departments can choose not to consult
Plot	A complex set of rules brings formal duties to generate smart results. Particularistic interests and capture can ruin the plot	Master plot and sub‐plots. Clearly defined steps taken in sequence lead to meaningful consultation—which in turns produces good policy and legitimacy	Consultation tools deployed in a context of governance innovations like the Legislative Footprint. High‐quality consultation creates the conditions for good governance and convergence with international good practice	The plot emphasizes the attractiveness of consultation. Through parables and edifying examples, officers generate the information they need
Moral	Evidence‐informed policy and legitimacy	Social legitimacy and trust achieved by dint of evidence‐based policy	Culture of innovation and openness	Primarily, outcomes benefit officers and government

There are also lessons about policy design. Consultation guidance is designed with few aggregation and payoff rules—revealing procedures that are likely to become ritualistic and symbolic unless there is political commitment at the top of the government. The narrations also show the diversity of government styles, from the Maltese case of internal dialogue between government and civil servant, Finland's pedagogies on inclusion, to the grandiose scenario of Principles in Ireland. The EU displays the DNA of an organization that, because of its democratic deficit, must operate within a web of nested norms that define a plethora of choice and information rules.
